# The Infant Version of the Laboratory Temperament Assessment Battery (Lab-TAB): Measurement Properties and Implications for Concepts of Temperament

**DOI:** 10.3389/fpsyg.2017.00846

**Published:** 2017-05-24

**Authors:** Elizabeth M. Planalp, Carol Van Hulle, Jeffrey R. Gagne, H. Hill Goldsmith

**Affiliations:** ^1^Department of Psychology, University of Wisconsin–MadisonMadison, WI, United States; ^2^Waisman Center, University of Wisconsin–MadisonMadison, WI, United States; ^3^Department of Psychology, University of Texas at ArlingtonArlington, TX, United States

**Keywords:** temperament, emotion, assessment, Lab-TAB, infancy

## Abstract

We describe large-sample research using the Infant Laboratory Temperament Assessment Battery (Lab-TAB; Goldsmith and Rothbart, 1996) in 1,076 infants at 6 and 12 months of age. The Lab-TAB was designed to assess temperament dimensions through a series of episodes that mimic everyday situations. Our goal is to provide guidelines for scoring Lab-TAB episodes to derive temperament composites. We also present a set of analyses examining mean differences and stability of temperament in early infancy, gender differences in infant temperament, as well as a validation of Lab-TAB episodes and composites with parent reported Infant Behavior Questionnaire (IBQ; Rothbart, 1981) scales. In general, laboratory observed temperament was only modestly related to parent reported temperament. However, temperament measures were significantly stable across time and several gender differences that align with previous research emerged. In sum, the Lab-TAB usefully assesses individual differences in infant emotionality.

## Introduction

Temperament is manifest in individual differences in emotional reactivity and regulation (Goldsmith et al., 1987; Goldsmith, 1993). As an early emerging set of behavioral tendencies, temperament is relatively stable through infancy and childhood, and forms a basis for later personality (Rothbart and Bates, 1998). Individual differences in infant temperament are conveniently assessed by parent report but more convincingly assessed objectively via elicited behavior in the laboratory or at home (Gagne et al., 2011). In this paper, we describe the use of the Laboratory Temperament Assessment Battery (Lab-TAB), an internationally used method to assess infant and child temperamental dimensions. We begin with a discussion of the definitional issues differentiating discrete emotion from temperament. Second, we highlight current and historical use of the Lab-TAB. Next, we describe each temperament dimension's observable components and inter-relations among them. Lastly, we briefly discuss existing research on gender differences and continuity in temperament dimensions across development.

### The distinction between emotional reactions and emotional traits (temperament)

Despite a long history of conceiving emotional differences as temperamental (Allport, 1937; Campos et al., 1983; Goldsmith, 1993, 1994), the distinction can be confusing. Here, we describe definitional differences between emotion and temperament from the perspective of Lab-TAB. Let us suppose that a single infant is tested in the Stranger Approach episode of the Lab-TAB and shows a fearful facial expression when the stranger first begins to approach. The infant's fearful facial expression should not necessarily be conceived as temperamental, but instead is an emotional reaction. What if the infant continues to show facial fear reactions throughout the Stranger Approach, is this *pattern* of facial expressions temperamental? Again, no. We have simply observed a series of facial expressions. Now, if 100 infants are tested using a Stranger Approach and we quantify their individual differences in patterns of facial expression, can we call these differences temperamental? Yes; temperament requires an individual differences perspective. However, our designation of these differences as temperament is simply definitional; it is open to dispute. If we now quantify individual differences in the sample to other responses that prove to be correlated with facial fear (such as withdrawal, freezing, looking away, and whimpering or crying) and infer a trait that accounts for correlated responses, we are on firmer ground for calling the pattern of reactions temperamental. Still, competing explanations for the correlated responses exist (e.g., individual differences in the effects of infant state, such as tiredness or hunger). Measured emotional reactions to other novel or threatening stimuli besides the stranger that converge across situation and extend over time (days, weeks, and longer) and demonstrate cross-situational consistency as well as longitudinal stability adds to our confidence that we are observing the manifestation of a temperamental trait, using Allport's (1937) definition. Individual instances of emotional behaviors are never properly viewed as temperamental; patterns of consistent and stable individual differences in emotion manifestation are temperamental, or at least they are consistent with temperament theory.

### Laboratory temperament assessment battery

At the most recent counting, we have received over 600 requests for the Pre-locomotor, Locomotor and Preschool Lab-TAB manuals from researchers in 36 different countries. We do not intend for Lab-TAB administration, coding or scoring to be prescriptive; therefore, researchers may use different coding schemes and modify administration of the episodes depending on their needs. Developmental scientists who have used the manuals over the last 25 years have addressed important questions about the early development of temperament and emotion from a multi-method perspective. For example, one group of researchers has used infant Lab-TAB data to examine the contextual basis of maternal perceptions of temperament (Hane et al., 2006), and another has examined interactions between negative temperament and parenting behaviors as risk factors for child obesity and obesity interventions (Anzman-Frasca et al., 2013, 2014). Others have examined early social and nonsocial fear and other temperament dimensions assessed in Lab-TAB episodes to predict the development of anxiety in early school years (Buss, 2011; Kiel and Buss, 2011; Brooker et al., 2016). Another group has noted remarkable stability in five temperament dimensions (Dyson et al., 2015) as well as an interaction between elevated cortisol levels and a high quality relationship with the primary parent predicting lower negative emotionality and higher positive emotionality in children from age three to six (Kopala-Sibley et al., 2017), using Lab-TAB assessments. However, psychometric properties of the infant Lab-TAB as a whole have not been examined in great depth or detail although we have examined properties of a home-administered version of Lab-TAB for older children, at age 4.5 years (Gagne et al., 2011). Although many investigators use one or a few Lab-TAB episodes as suitable for their research (e.g., Braungart-Rieker et al., 2010; Degnan et al., 2011; He et al., 2013), a need remains for a comprehensive examination of multiple dimensions of infant temperament to garner a more holistic, person-centered understanding of infant emotional development.

### Temperament dimensions

Following an intellectual tradition dating back to Darwin (1872), discrete emotion theorists such as Tomkins (1982), Ekman (1982), Izard (1972), Izard and Malatesta (1987), and Plutchik (1980) identify fear, anger, sadness, joy, interest, disgust, and surprise as fundamental emotions, along with other feeling states (Ekman, 1982, 1989). Temperament theorists traditionally include activity level as aspects of temperament (Rothbart, 1981; Goldsmith and Campos, 1986) and sometimes point out affective features of this overtly non-emotional temperament construct (Goldsmith and Campos, 1986; Goldsmith et al., 1987). For the purposes of this paper, we examine individual differences in fear, anger, sadness, positive affect, interest/persistence, and activity level.

Although researchers often use a more general measure of negative affect in early infancy, we believe that a broad dimension of proneness to distress is less useful than more focal dimensions of fearfulness, anger proneness, and sadness, which show differences in function, expression, and development (Campos et al., 1983; Clifford et al., 2015). Previous research (Braungart-Rieker et al., 2010; Clifford et al., 2015) has indicated etiological as well as behavioral differences in fear, anger and sadness. Though all conceptually negative, anger is related to approach behaviors whereas fear and sadness are related to withdrawal behaviors. Fear is typically expressed during novel situations whereas sadness is expressed upon loss of an object or need. Thus, evidence from outside Lab-TAB dictates that these three dimensions represent different aspects of hedonic negativity and thus should be examined separately.

#### Fearfulness

Fear is sometimes exhibited when the infant responds to stimuli that convey the threat of harm or uncertainty, for example, a novel or unfamiliar person. Infant fear is expressed with behaviors such as crying, freezing, or escape. The associated action tendency is withdrawal or avoidance. Fear can be an evolutionarily adaptive mechanism used to protect oneself, and behavioral expressions of fear during infancy can elicit aid from caregivers (Campos et al., 1983; Buss and Goldsmith, 1998). Fear is often grouped with anger and sadness as components of an overall negative affectivity factor (Goldsmith, 1996; Putnam et al., 2001). In addition, fear in infancy predicts behavioral inhibition, the tendency to express distress and withdraw in the face of novelty or uncertainty, in early childhood (Kagan, 1997; Brooker et al., 2013). The individual differences facet of fear is regarded as temperamental.

#### Anger

As a temperament dimension, anger/frustration is the child's propensity toward expressing a negative affective response within a challenging situation, such as contexts where attack or goal blockage is perceived (Stenberg and Campos, 1990; Gagne et al., 2009). The action tendency for anger is approach (as opposed to fear or sadness), and relevant behaviors are crying, protesting, hitting or pushing, and pouting (Goldsmith and Rothbart, 1991; Buss and Goldsmith, 1998). Anger and frustration are evident early in infancy and considered normative markers of development (Stenberg et al., 1983).

#### Sadness

Sadness is considered a primary emotion (Izard and Malatesta, 1987), yet it is often included in an overall negativity factor rather than examined independently as a dimension of temperament (Putnam et al., 2001). Like anger, sadness reflects an infant's reaction to goal blockage or loss, but without an approach orientation. Sadness is expressed with crying, pouting, and at times a slumped head and/or shoulders. Sadness also shares a withdrawal action tendency with fearfulness, without the novel or threatening incentive features associated with fearfulness.

#### Positive affect

Positive affect is relatively neglected in the literature compared with hedonically negative aspects of infant temperament (Gartstein and Marmion, 2008). Positive affect is exhibited with expressions of laughter, smiling, and certain motor behaviors and vocalizations (Campos et al., 1983). Signs of positive affect appear as early as 2 months of age, when infants start to indicate joy with smiling and positive vocalizations (Sroufe and Waters, 1976). From an evolutionary perspective, the social smile becomes an integral component of socioemotional development, with infants and caregivers utilizing smiling to enhance attachment bonds (Sroufe and Waters, 1976). Similar to anger, positive affect is an approach-related behavior, relating to activity level and impulsivity in childhood (Rothbart et al., 2000; He et al., 2013).

#### Interest/persistence

Infant interest or persistence, operationalized as duration of orienting by Rothbart (1981), refers to an infant's tendency to look at or play with an object for an extended period of time in the absence of obstacles or interruptions. Interest/persistence reflects the motivational system underlying many attentional systems and the notion of individual differences in the deployment of interest/persistence is often included as part of a broader dimension such as effortful control (Rothbart and Goldsmith, 1985) or task orientation (Matheny, 1980). These measures show stability across infancy (Rothbart et al., 2000; Auerbach et al., 2004) but should not be confused with the cognitive construct of visual attention used to mark the development of executive function (Colombo, 2002). Indeed, the development of an executive attention system may underlie effortful control (Posner et al., 2016). Gartstein and Rothbart (2003) found that interest and attentional orienting measures cluster with positive affect, but other studies find no relationship between interest, attentional orienting and other temperament dimensions (Matheny, 1980; Rothbart, 1981). Despite its potential role in predicting key developmental milestones and its status as complex set of systems that form a cornerstone of cognitive development, infant interest/persistence is omitted from studies of infant temperament.

#### Activity level

Activity level, although not overtly emotion-related, is a traditional temperament dimension (Escalona, 1968). Activity level refers to the typical level of energy expenditure through gross motor movements (Saudino, 2012); this energy expenditure often occurs in the service of attaining goals, much as more emotion-related features of temperament motivate individuals to attain goals. In infancy, activity level can be operationally defined as individual differences in proneness to higher or lower degrees of intensity and frequency of movement of the arms, legs, head, or trunk (Gagne et al., 2009). In general, infants who are more active are also higher on approach based temperamental tendencies such as anger and exuberance (He et al., 2013) and show less sustained attention in toddlerhood (Goldsmith, 1996).

### Assessment of infant temperament

The idea that temperament dimensions manifest in a diverse set of behaviors is well-established. More discrete and quantitative measurement of these dimensions in infancy becomes difficult because we must depend entirely on behavioral observations without the benefit of knowing with certainty whether the infant interprets the context of assessment, including the affective incentives, in the way that investigators intend. Temperament measures can take many forms: parent reports of infant's responses to various scenarios or laboratory assessment of infant reactions to specific stimuli are often utilized to assess infant behaviors (Rothbart and Goldsmith, 1985). Despite some convergence across parent reported and laboratory assessed infant behaviors, the differences between these two modes of assessment have been explicated extensively (Goldsmith and Rieser-Danner, 1990). Among the key differences are that parents generalize across contexts and time and bring their own biases to bear in interpreting behavior whereas laboratory measures reflect objective, minimally interpreted responses to a specific context over period as short as a minute. Thus, the lack of strong convergence is unsurprising.

### Gender differences in temperament

The magnitude of gender differences in toddler and child temperament appears to be modest and variable depending on which dimension of temperament is under investigation (Chaplin and Aldao, 2013). A recent meta-analysis of gender differences in temperament indicated that boys tend to be higher in approach based tendencies such as anger, exuberance, and activity level whereas girls are higher in shyness, some fear behaviors, and inhibitory control (Else-Quest et al., 2006). Our results in this paper further examines this issue in the pursuit of describing and validating a laboratory based assessment of infant temperament.

### Continuity of temperament in infancy

Emotional behaviors vary throughout development; early in infancy, we generally conceive of emotional behaviors as solely reactive in nature. The variety of these reactive behavior increases during infancy, and their solely reactive nature also changes. From 6 to 12 months of age (the age period examined empirically in this paper), infants grow rapidly. During this period, infants develop more muscle tone and the ability to stabilize themselves and sit more easily; they begin to crawl and then to pull themselves to standing, and finally begin to walk. These advances in motor control align with cognitive and motivational changes and support an expanding repertoire of emotional expressions (Izard and Malatesta, 1987). Moreover, increased motor and cognitive control allows infants to better regulate emotions as they approach 12 months of age (Thompson, 1991). In other words, infants develop more ways to express emotion concurrently with developing ways to downregulate emotions—especially hedonically negative emotions—more effectively. Temperament theorists generally expect that individual differences in the tendency to respond to emotionally laden stimuli remain relatively stable across infancy (Goldsmith et al., 1987; Rothbart and Bates, 1998).

### Research questions

In a sample of 6 and 12 month infants, we discuss scale development and scoring of tasks that elicit the primary emotions and describe gender differences and stability in infant temperament. Further, we use multiple methods (parent report and laboratory-based, elicited behaviors) to examine relations across reporter. We address the following questions: (1) How do we construct scores for laboratory assessments of infant temperament? (2) What degree of convergence exists between laboratory and parent report infant temperament? (3) To what extent does gender relate to infant temperament, and are temperament dimensions stable from 6 to 12 months?

## Methods

Data were drawn from a longitudinal study of twins examining the development of infant temperament/emotionality. In accordance with the university's Institutional Review Board, families were recruited using multiple methods, including state birth records, newspaper birth announcements, television advertising, and flyers in doctors' offices. See Lemery-Chalfant et al. (2006) for sampling and details regarding recruitment methods.

### Participants

Parents and their infant twins (*N* = 1,076 infants) were assessed in both the laboratory and via questionnaire methods. Some 92.8% of infants were Caucasian (3.2% Hispanic), 3.5% of infants were African-American, and 1.7% were Asian-American. Approximately half the infants (*n* = 550, 51.5%) were female. Mothers were on average 32 years old (*M* = 31.86, *SD* = 4.79) and fathers were 34 years old (*M* = 33.67, *SD* = 5.68), with median family income above $50,000. Seventy-five percent (75.4%) of mothers and 77.1% of fathers had completed college and 14.9 and 20.6% of mothers and fathers, respectively, had a high school degree.

### Procedures

Infants participated in videotaped laboratory assessments of temperament at 6 and 12 months of age. Mothers brought their infants into the lab and completed the Lab-TAB with each infant tested in a tandem fashion (i.e., separate testing of each twin, conducted in a multi-room laboratory with a large team, such that co-twins could be tested at the same time). Following the laboratory visit, mothers and fathers completed questionnaires measuring infant temperament. This study was carried out in accordance with the recommendations of the University of Wisconsin-Madison's Institutional Review Board with written informed consent from all subjects.

### Measures

#### Infant behavior questionnaire

Parent reported infant temperament was measured at 6 and 12 months using the Infant Behavior Questionnaire (IBQ; Rothbart, 1981). The IBQ is one of the most widely used measures of infant temperament. Composed of six scales, it asks parents to rate the frequency of their infants' behaviors (crying, smiling, looking, etc.) across a variety of situations, for example while feeding, bathing, or changing. By asking parents to rate their infant's behavior during specific situations, the IBQ seeks to minimize global perceptions and interpretations of infant temperament (Rothbart and Goldsmith, 1985). Instead, parents remember discrete instances in which their infants acted in a specific way and respond to the IBQ items accordingly. Further, in our study each parent reported on each twin's behavior. The granular nature of IBQ items prevents parents from inflating behavioral differences in their offspring (Goldsmith et al., 1997). Therefore, scales derived from the IBQ items are intended to represent each individual infant's pattern of responding across specific situations.

The IBQ measures temperament using 6 scales: Fear, Distress to Limitations, Smiling, and Laughter, Soothability, Duration of Orienting, and Activity Level. Mothers and fathers rated infant behavior on a seven-point Likert scale, ranging from “never” to “always,” for 94 questions. Internal consistency was calculated for mother and father reports on the IBQ scale at 6 and 12 months and are presented in **Table 2**. Additionally, mother and father reports were significantly correlated for each scale (see **Table 2**); thus, we created a composite score for infant temperament using an average of mother and father reports. The IBQ Distress to Limitations and Duration of Orienting scales are comparable to Lab-TAB measures of Anger and Interest/Persistence.

#### Laboratory temperament assessment battery

The Laboratory Temperament Assessment Battery (Lab-TAB; Goldsmith and Rothbart, 1991) assesses infant responses to stimuli in a controlled setting and is a standardized way to elicit and score behaviors. Many adult emotion coding systems rely on facial expressions (i.e., AFFEX; Izard and Malatesta, 1987), but infant emotion is also expressed through bodily movements (Weinberg and Tronick, 1994) and vocalizations, which are incorporated in the Lab-TAB coding system. Although numerous studies examine infant emotion using behavioral coding (e.g., Morris et al., 2011), no study focuses on validating the psychometric properties of the Lab-TAB in infants.

Lab-TAB episodes are standardized and designed to measure infant's reactions to stimuli which elicit emotional or behavioral reactivity across five broad dimensions of infant temperament: fearfulness, anger proneness, joy/pleasure, interest/persistence, and activity level. Although the Lab-TAB manual describes 20 episodes, researchers may choose and implement those which best support their study design. At each age, we used two episodes measuring fear, anger, sadness, positive affect, and one episode each for activity level and interest taken from the Pre-Locomotor and Locomotor versions (Goldsmith and Rothbart, 1996). A complete description of procedures and coding is in the Lab-TAB manuals. Visits were videotaped for later scoring. Coders were trained by a highly experienced coder and kappas for scorer agreement on each episode are described below. We give brief episode descriptions and dimensions measured in Table 1.

**Table 1:** **IBQ scales and Lab-TAB episode descriptions and scoring components**.

**IBQ Scale**	**Temperament dimension**	**Description**	
Fear	Fear	Infant's response to novel physical objects or social stimuli; inhibited approach to novelty	
Distress to limitations	Anger	Infant's fussy, crying, distress behaviors with confined in place or position, involved in caretaking situations, or inability to perform a desired action	
Smiling and laughter	Positive affect	Smiling and laughter by the infant in general caretaking and play situations	
Soothability	Regulation	Infant's response to soothing techniques by caregiver when the infant is fussy, crying, or in distress	
Duration of orienting	Regulation	Infant's attention to and/or interaction with an object for extended periods of time	
Activity level	Activity level	Infant's gross motor activity, movement of arms, and legs, squirming, locomotor activity	
**Lab-TAB episode**	**Temperament dimension**	**Description**	**Episode-level scoring components**
Stranger approach	Fear	Social interaction with an unfamiliar male stranger	Facial and bodily fear, facial, and bodily sadness, distress vocalizations, escape behaviors
Masks	Fear	Non-social fear at the appearance of several relatively mild, non-intrusive novel masks	Facial and bodily fear, facial, and bodily sadness, distress vocalizations, escape behaviors
Arm restraint	Anger, sadness	Prevent the child from playing with a novel, interesting toy	Bodily struggle, facial anger, distress vocalizations, bodily sadness, facial sadness
Car seat	Anger, sadness	Restrain child in an unfamiliar car seat	Bodily struggle, facial anger, distress vocalizations, bodily sadness, facial sadness
Puppets	Positive affect	Pleasure toward puppets in a social play game	Smiling, laughter, positive vocalizations and positive motor acts, smiling intensity
Peekaboo	Positive Affect	Pleasure toward mother in a social stimulation	Smiling, laughter, positive vocalizations and positive motor acts, smiling intensity
Slides	Interest/persistence	Expression of interest in a non-social context	Facial interest, duration of looking, presence of gestures, presence of vocalizations
Basket of Toys (6 months only)	Activity level	Activity level during object-oriented play	Intensity of toy manipulation, number of bouts of toy play
Free play (12 months only)	Activity level	Activity level when several toys are available for play	Intensity of toy manipulation, number of bouts of toy play, intensity of overall movement, changes in body position.

*IBQ, Infant Behavior Questionnaire; Lab-TAB, Laboratory Temperament Assessment Battery*.

##### Fear: stranger approach

During Stranger Approach the child is placed in a high chair across the room from a door where a stranger (i.e., male tester unknown to infant) enters. The mother is also in the room but instructed not to interact with the infant. The Stranger Approach occurs in several stages. First, the stranger enters the room and waits for 10 s. Then the stranger slowly moves toward the infant stops approximately halfway into the room (10 s), saying “Hello [infant's name]. I'm going to come a little closer to you now.” The stranger slowly walks close to the infant (10 s) and once the stranger arrives at the infant's location, he kneels down and stares at the infant for 30 s. Behaviors are scored by trained coders in three stages: the stranger's entry (10 s), the stranger's slow approach (20 s), and the stranger staring at the infant 30 s. This 30 s is broken into 6 5-s scoring epochs. Scores for each stage include facial and bodily fear (0–3 scale), facial sadness (0–3 scale) and bodily sadness (0/1), distress vocalizations (0–5 scale), and escape behaviors (0–3 scale). Kappas assessing the reliability of coding ranged from 0.71 to 0.90, with an average κ = 0.80.

##### Fear: masks

For the Masks episode, the infant is presented with four increasingly scary masks for 10 s each. Each infant is presented the same masks in the same order; first, an evil cartoon queen, second, an old man, third, a green vampire mask, and lastly a gas mask. Each “mask” trial is then divided into two scoring epochs of 5 s. Several behaviors are scored during each trial: facial and bodily fear (0–3 scale), facial (0–3 scale) and bodily (0/1) sadness, distress vocalizations (0–5 scale), and escape behaviors (0–3 scale). Reliability estimates for Masks ranged from 0.68 to 0.87, with an average κ = 0.76.

##### Anger and sadness: overview

Infant response to goal blockage is often assessed by using laboratory measures of restraint, where the infant is unable to access something he/she wants, namely an attractive toy or free movement. From these tasks, we code an infant's facial, vocalic, and behavioral reactions reflecting both anger and sadness.

*Anger and sadness: arm restraint*. In the Arm Restraint episode, infants are shown an enticing perpetual motion toy and allowed to play with it until the mother restrains the infant arms so the infant cannot touch or play with the toy. This is repeated twice resulting in two 30 s trials. Each trial is then divided into 6 5-s epochs from which we score bodily struggle (0–4 Likert scale), facial anger and facial sadness (0–3 scale), distress vocalizations (0–5 scale), and bodily sadness (0/1). Average reliability for Arm Restraint was κ = 0.79 (range from κ = 0.69 to κ = 0.83).

*Anger and sadness: car seat*. In the Car Seat episode, the infant is placed in a car seat by the parent and physically restrained for a total of 30 s. Like Arm Restraint, we code the presence or intensity of facial anger (0–3 scale), bodily anger or struggle (0–4 scale), and distress vocalizations (0–5 scale) every 5 s. Facial sadness (0–3 scale) and bodily sadness (0/1) are also scored during the Car Seat episode. Scoring reliability estimates for Car Seat ranged from 0.63 to 0.93, with an average κ = 0.77.

##### Positive affect: puppets

For the Puppets episode, the experimenter wears two fun hand puppets and uses them to engage the infant in game playing. Using voices for each of the puppets the experimenter talks to the infant, tickles the infant, and then allows the infant to play with the puppets over the course of 2 min. The first 90 s are divided into 4 scoring epochs. The first starts upon the emergence of the puppets. The second, third, and fourth epochs begin each time the experimenter tickles the infant with the puppets. Finally, the fifth 30 s epoch starts when the experimenter lays the puppets on the table in front of the infant to see if the infant wants to play with them. Scoring is completed for each epoch and several variables are scored based on infant behaviors with or toward the puppets. Smiling, laughter, positive vocalizations and positive motor acts are scored as 0 “not present and 1 “present.” Smiling intensity is scored on a Likert scale ranging from 0 “no smiling” to 3 “large smile.” Scoring reliability estimates for Puppets ranged from 0.80 to 0.89, with an average κ = 0.85.

##### Positive affect: peekaboo

In the Peekaboo episode, mothers engage the child in a game of peekaboo by standing behind a screen. Over the course of six trials, the experimenter prompts the child with “Where's Mommy? Where did she go?” and then opens one of three doors in a wooden screen. For the first three trials as well as the last trial the mother is behind the screen and says “Peek-A-Boo!” For trials four and five, the mother is not behind the screen and the experimenter says “Oops, not there.” The entire episode lasts approximately 1 min. As in the Puppets episode, several items are scored for each of the six trials: smiling, laughter positive vocalizations and positive motor acts (0/1) and intensity of smiling (0–3 scale). Scoring reliability for Peekaboo had an average κ = 0.86, with a range from κ = 0.84 to 0.92.

##### Interest/persistence: slides

Laboratory assessments of interest typically measure how long an infant gazes at or manipulates an object in an unstructured task. The infant is oriented toward a screen where he/she views a series of slides depicting children, mothers and their children, and various nature scenes. The infant views 5 slides in three 30-s trials for a total of 15 slides. The first slide is presented for 2 s (Epoch 1), the second slide is presented for 4 s (Epoch 2), the third slide is presented for 6 s (Epoch 3), the fourth slide is presented for 8 s (Epoch 4) and finally the last slide of the trial is presented for 10 s (Epoch 5). This is repeated three times to total 1.5 min. From this, we scored facial interest (0–2 scale), duration of looking (0–3 scale), and the presence of gestures (0/1) and vocalizations (0/1). Scoring reliability estimates for Slides ranged from 0.79 to 0.88, with an average κ = 0.85.

##### Activity level: basket of toys

During Basket of Toys (6 months only), the infant is placed beside a basket filled with enticing toys, such as a rattle or stuffed animal. The infant's seating is supported and coders observe the patterns of toy play for 3 min. This is divided into 9 20-s scoring epochs. Coders score the intensity of the infant's manipulation of the toys (0–4 scale) and the number of different toys the infant plays with during each epoch.

##### Activity level: free play

During the Free Play episode (12 months only), children are allowed to move around a room filled with toys, such as a truck, balls, inflatable rings, and a drum. The toys are set up in a circle and the child is left to play with the toys for 6 min. The first minute is a warm-up and is not coded. The remaining 5 min are divided into 15 20-s scoring epochs. By 12 months old, infants have more dexterity and are more mobile, so in addition to the intensity of manipulation of toys (0–4 scale), and number of toys the infant plays with, we also score intensity of movement (0–4 scale) and number changes in body position. Scoring Kappas for the activity level composites ranged from 0.60 to 0.80, with an average κ = 0.71.

### Missing data

Recruitment was ongoing throughout the course of the study; thus, more families participated in the 12-month than 6-month assessment (see Table 2 for *N*s, which reflect both the continuing recruitment and missing data). Analyses used full information maximum likelihood estimation (FIML; Enders, 2010), a robust method for approximating unbiased parameter estimates by utilizing all available data.

**Table 2:** **Internal consistency and cross-parent (mother-father) for IBQ scales and Lab-TAB infant temperament episodes**.

	**6-month**				**12-month**			
**Temperament measure**	***N***	**Mother α**	**Father α**	**Cross-parent correlation *r***	***N***	**Mother α**	**Father α**	**Cross-parent correlation *r***
IBQ Fear	500	0.79	0.74	0.41^***^	709	0.77	0.79	0.57^***^
IBQ distress to limitations	494	0.83	0.84	0.51^***^	705	0.83	0.82	0.46^***^
IBQ smiling and laughter	500	0.80	0.84	0.31^***^	713	0.81	0.82	0.38^***^
IBQ soothability	499	0.68	0.91	0.18^**^	713	0.66	0.63	0.11^**^
IBQ orienting	500	0.82	0.80	0.27^***^	713	0.83	0.84	0.36^***^
IBQ activity level	500	0.83	0.84	0.49^***^	713	0.82	0.81	0.31^***^
			α				α	
Fear—stranger	515		0.74		802		0.74	
Fear—masks	557		0.70		878		0.77	
Anger—arm restraint	537		0.90		826		0.90	
Anger—car seat	499		0.90		775		0.87	
Sadness—arm restraint	536		0.65		825		0.64	
Sadness—car seat	499		0.72		775		0.72	
Positive Affect—puppets	551		0.81		867		0.82	
Positive Affect—peekaboo	534		0.82		833		0.86	
Slides/interest	536		0.82		877		0.77	
Activity Level (BT and FP)	369		0.88		837		0.77	

Note:

* p < 0.05;

** p < 0.01;

*** p < 0.001;

*IBQ, Infant Behavior Questionnaire; Lab-TAB, Laboratory Temperament Assessment Battery; BT, Basket of Toys; FP, Free Play. In each case; Lab-TAB composites included the mean and peak of each behavior. Correlations account for non-independence of twins within family*.

## Results

First, we describe data reduction methods for each of the Lab-TAB episodes. Second, we examine measures of reliability of each temperament dimension tapped by the IBQ and Lab-TAB at both 6 and 12 months of age. Then, we examine stability from 6 to 12 months. Fourth, we analyze the degree of convergence of each temperament dimension across parent and observed assessments. Lastly, we examine gender differences.

### Data reduction for the LAB-Tab episode and dimension composites

**Figure 1:**
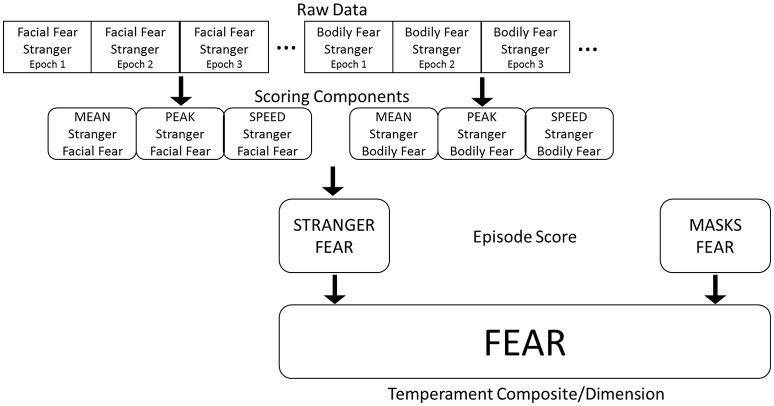
**Data reduction to derive temperament composites from Lab-TAB raw data**.

The Lab-TAB yields several scores (e.g., facial affect ratings) for each epoch (intervals of a few seconds) across each trial within each episode of the protocol. The resulting amount of raw data from as few as 10 episodes can be overwhelming. Here, we describe a standardized procedure for scoring the Lab-TAB and reducing the data to more useable episode-level variables for each desired construct. Figure 1 depicts our data reduction strategy to derive temperament composites from Lab-TAB raw data.

The first step in data reduction is to create response parameters for behaviors across the episode. For example, in the Masks episode the infant receives a raw score for facial fear on each epoch (*n* = 2) for each mask (*n* = 4), resulting in 8 scores. Thus, we average facial fear across the 8 epochs and use this as a mean facial fear score for Masks. By examining the mean level of each behavior that the infant exhibits, we quantify the infant's average reactivity for each episode. Consequently, shorter latencies to show facial fear, longer durations of facial fear, and higher levels of facial fear will all be reflected in higher means. Next, we determine the peak level of facial fear for the episode, whenever this peak might have occurred during the 8 epochs. Peaks and means tend to be correlated, but peaks better capture an infant's tendency to respond to stimuli using extreme behavioral responses. Two infants may have the same mean facial fear scores, but very different peak scores.

Finally, we calculate the latency, or the time before, the occurrence of a behavior. We do so indirectly by counting the number of epochs before the first occurrence of facial fear, for example. If facial fear is apparent right away, then the latency it takes to observe it is 0 because it occurs in the first epoch. If facial fear is not present until the third mask, the latency is 4 because it occurs in the fifth epoch. We then reverse score these latencies to reflect the speed with which the infant exhibited the behavior. This procedure results in mean, peak, and speed scores that are all in the same direction (i.e., higher scores imply more intense or frequent occurrence of a behavior) and tend to be correlated within an episode. We suggest that researchers follow this approach initially then use the mean, peak and speed scores as best fits their research question.

For this paper, we combined mean and peak scores across trials within each episode to create an overall episode score. Factor analyses to determine which scoring components to include in each episodic score and then reliability statistics (Cronbach's alphas) were calculated for each episode at each age. The speed scores did not add significant variation to our episode-level scoring, and these speeds are therefore not included in further analyses. Researchers who are interested in examining how quickly an infant may react to a stimulus, or more generally in examining emotion expression using a chronometric approach (Davidson, 1998) may opt to include the speed scores as well. The resulting episode-level scores include each of the scoring components listed in Table 1. For the fear episodes, escape behaviors and bodily fear (freezing) are mutually exclusive behaviors, so only bodily fear is included in the Stranger Approach and Masks episode-level scoring. We also combined scores across episodes to create an overall score for each temperament dimension of interest. For example, Masks and Stranger Approach both assess infant fear so we combined these for an overall Fear composite (see Figure 1).

Table 2 provides the reliability statistics for the episode-level and composite scores derived from each Lab-TAB episode. For the IBQ scales, alphas were satisfactory and generally consistent with the literature. The IBQ Soothability scale tended to somewhat less internally consistency than the other scales, possibly because the nature of the items on this scale ask about the effectiveness of different soothing techniques. High internal consistency would require most techniques to be equally effective, which may not be the case. Mother-father correlational agreement on all IBQ scales was significant at both ages in this large sample. The magnitude of the agreement might be characterized as modest-to-moderate (correlations in the 0.30–0.50 s), except for Soothability.

Turning to the Lab-TAB (lower portion of Table 2), internal consistency estimates averaged 0.794 at 6 months and 0.786 at 12 months. Variation in internal consistency across composites was limited, with Anger measures tending to be highly consistent and Sadness measures somewhat less so. Although internal consistency was enhanced by the composite construction process and would be lower in a replication sample, we judged these levels of internal consistency as satisfactory.

We created an SES variable measuring mother and father education as well as family income. SES was inversely related to parent reported IBQ Fear at 6 and 12 months of age as well as IBQ Anger and at 12 months of age. It is possible that lower SES parents encounter more external stressors which affect their reporting of infant behaviors. Only one significant relation emerged between SES and observed Lab-TAB temperament (during Basket of Toys at 6 months of age, *r* = 0.13, *p* < 0.01); thus, we did not include SES in further analyses.

### Parent reported infant temperament

Inter-correlations of the IBQ scales are presented in the upper left portions of Table 3 (6 months) and 4 (12 months). These correlational patterns align with previous literature examining the IBQ (Putnam et al., 2001). Scales assessing infant negativity, namely Fear and Anger, were significantly correlated with each other at 6 and 12 months. Scales assessing infant regulatory behaviors (Soothability and Duration of Orienting) were also significantly related at each age. Smiling and Laughter was positively correlated with Soothability and Duration of Orienting at both ages. Only at age 12 months was the most hedonically positive scale (Smiling and Laughter) negatively correlated with the hedonically negative Fear and Anger scales. Finally, infants who were higher in Activity Level also expressed more emotionally driven behaviors, as reflected in the Fear, Anger and Smiling and Laughter scales at each age.

**Table 3:** **Correlations between 6-month temperament measures**.

**Temperament measure**	**1**	**2**	**3**	**4**	**5**	**6**	**7**	**8**	**9**	**10**	**11**	**12**	**13**	**14**	**15**	**16**	**17**	**18**	**19**	**20**
IBQ fear	1																			
IBQ distress/anger	**0.10**	1																		
IBQ smiling	−0.07	−0.02	1																	
IBQ soothability	0.05	−0.04	**0.30**	1																
IBQ orienting	0.05	−**0.11**	**0.27**	**0.25**	1															
IBQ activity	**0.17**	**0.40**	**0.21**	0.03	0.01	1														
Fear—stranger	0.02	0.02	−0.05	−0.05	0.06	0.10	1													
Fear—masks	0.04	0.04	0.05	−0.04	0.04	0.04	**0.15**	1												
Anger—AR	−0.01	**0.35**	0.02	0.02	−0.05	−**0.15**	0.02	**0.20**	1											
Anger—CS	**0.27**	−0.04	0.03	0.10	−0.12	0.06	**0.19**	**0.27**	**0.28**	1										
Sadness—AR	0.05	−**0.27**	−0.13	−0.06	−0.07	**0.24**	0.04	**0.19**	**0.63**	**0.20**	1									
Sadness—CS	−**0.22**	**0.23**	0.01	−0.09	0.10	−0.09	**0.19**	**0.24**	**0.13**	**0.52**	**0.29**	1								
PA—puppets	0.02	0.00	0.16	−**0.17**	0.05	0.00	−**0.12**	−0.06	0.04	−0.03	0.03	−0.04	1							
PA—peekaboo	−0.10	0.01	0.15	−0.01	−**0.16**	0.09	−0.06	0.01	0.03	−0.01	−0.02	0.03	**0.28**	1						
Slides/interest	0.01	0.02	0.00	−0.07	0.01	0.04	−0.03	0.05	−0.04	−0.05	0.00	0.04	−0.07	**0.10**	1					
AL—BT	0.04	−0.09	0.11	0.11	−0.05	0.10	0.11	0.08	**0.11**	0.11	**0.16**	**0.33**	**0.22**	**0.21**	0.01	1				
Lab-TAB fear	0.05	0.02	0.03	−0.09	0.06	0.13	–	–	0.03	**0.25**	**0.18**	**0.13**	−**0.12**	−0.01	−0.03	0.04	1			
Lab-TAB anger	**0.30**	−0.01	0.01	0.12	−0.15	0.06	**11**	**0.21**	–	–	**0.44**	**0.31**	0.01	0.03	−**0.09**	−**0.09**	**0.24**	1		
Lab-TAB sadness	−**0.23**	0.21	−0.01	−0.08	0.07	−0.03	0.05	0.09	**0.32**	**0.39**	–	–	−0.01	−0.01	0.04	**0.21**	**0.26**	**0.55**	1	
Lab-TAB PA	−0.02	−0.03	**0.27**	−**0.13**	−0.12	0.09	−**0.14**	−0.04	0.02	0.02	−0.01	0.00	–	–	0.01	**0.29**	−**0.11**	0.01	−0.02	1

*Bold correlations are significant at the 0.05 level. Ns range from 380 to 557. Due to varying sample sizes, the size of the correlation required to reach significance varies for each scale or episode. IBQ, Infant Behavior Questionnaire; AR, Arm Restraint; PA, Positive Affect; AL, Activity Level; BT, Basket of Toys; Lab-TAB, Laboratory Temperament Assessment Battery. Correlations account for non-independence of twins within family*.

### Observed infant temperament

Correlations within the Lab-TAB episodes and dimensions are shown in the lower right portions of Table 3 (6 months) and 4 (12 months). The first result is that our strategy for creating cross-episode composites was supported although the magnitude of cross-episode convergence was usually modest. Episodes were significantly correlated (all *p*s < 0.01) with the other episode within each dimension at each age: Stranger Approach and Masks (fear) scores were related at 6 (*r* = 0.15) and 12 months (*r* = 0.18); Arm Restraint and Car Seat Anger were related at 6 (*r* = 0.28) and 12 months (*r* = 0.17); Arm Restraint and Car Seat Sadness were related at 6 (*r* = 0.29) and 12 months (*r* = 0.27); and finally, Puppets and Peekaboo were related at 6 (*r* = 0.28) and 12 months (*r* = 0.28). Thus, we created Lab-TAB dimensions for Fear, Anger, Sadness, and Positive Affect at each age.

In general, episodes within the negative temperament dimension were significantly intercorrelated (Stranger Approach, Masks, Arm Restraint, Car Seat). This pattern was also reflected in the relations among the composites, such that Fear, Anger, and Sadness were consistently related to each other at 6 and 12 months. Activity level was related to Anger, Sadness and Positive Affect episodes at each age as well. Further, Positive Affect was negatively correlated with Anger at 6 and 12 months.

### Relations between IBQ and LAB-TAB

Associations between conceptually related questionnaire and observational measures speak to criterion-oriented validity, and those associations were not strong, generally speaking. Parent reported infant fear was related to Car Seat anger and sadness at 6 months (but not the expected fear measures from Lab-TAB), but by 12 months of age parent rated fear was correlated with laboratory measured fear, anger, and sadness. Parent reported distress/anger was modestly correlated, in most but not all cases, with increased anger and decreased sadness in the laboratory at both 6 and 12 months. Parent reported smiling/positivity was unrelated to any laboratory assessed temperament at 6 months, but by 12 months it was related to increased anger and decreased sadness during the Arm Restraint episode. Parent rated infant regulation, namely the Soothability and Orienting scales, were inversely related to laboratory positive affect at each age, but increased fear and interest and decreased anger and sadness at 12 months.

### Stability and gender differences in temperament across time

Our third aim was to examine stability of temperament across time, and determine whether this was similar for boys and girls. Table 5 lists the mean levels of each parent reported and laboratory observed temperament dimension by age and gender. Parents reported girls to be very modestly higher in Fear at both 6 (Cohen's *d* = 0.21) and 12 months (Cohen's *d* = 0.15), which was a salient finding, given no other gender differences for parent reported temperament. A similar trend occurred for observed fear for the Stranger and in the Masks episodes of the Lab-TAB although the differences between boys and girls did not reach statistical significance (*p* = 0.06). However, when Lab-TAB episodes were combined to create an overall observed fear composite, girls exhibited more fear than boys at 6 months but not 12 months.

**Table 4:** **Correlations between 12-month temperament measures**.

**Temperament measure**	**1**	**2**	**3**	**4**	**5**	**6**	**7**	**8**	**9**	**10**	**11**	**12**	**13**	**14**	**15**	**16**	**17**	**18**	**19**	
IBQ fear	1																			
IBQ distress/anger	**0.32**	1																		
IBQ smiling	−**0.22**	−**0.10**	1																	
IBQ soothability	−0.02	−0.02	**0.37**	1																
IBQ orienting	0.05	−0.10	0.**29**	**0.23**	1															
IBQ activity	**0.19**	**0.40**	0.12	0.07	0.07	1														
Fear—stranger	**0.14**	−0.03	0.04	0.04	−0.09	0.01	1													
Fear—Masks	**0.11**	0.01	−0.03	0.00	**0.10**	0.01	**0.18**	1												
Anger—AR	**0.25**	0.02	**0.28**	−0.04	−**0.12**	0.01	0.01	−0.07	1											
Anger—CS	−0.04	**0.30**	−0.02	0.00	0.06	−0.03	**0.10**	−0.03	**0.17**	1										
Sadness—AR	−**0.22**	0.08	−**0.21**	0.05	0.10	−0.03	**0.14**	0.06	**0.60**	**0.09**	1									
Sadness—CS	0.08	−**0.20**	−0.01	0.07	−**0.22**	0.09	**0.11**	**0.08**	**0.09**	**0.48**	**0.27**	1								
PA—puppets	−**0.10**	0.01	0.02	0.08	0.00	**0.12**	−**0.11**	−0.04	**0.09**	−0.01	−0.04	−0.07	1							
PA—peekaboo	−0.03	0.04	0.03	−0.01	0.03	−0.05	−0.06	−**0.08**	**0.15**	−0.01	0.02	0.03	**0.28**	1						
Slides/interest	0.06	−0.05	0.01	−0.05	**0.13**	0.01	−**0.09**	−0.02	−0.02	−**0.12**	−0.05	−0.06	**0.09**	**0.07**	1					
AL–FP	−0.05	.09	0.04	0.00	−0.06	0.07	0.01	−0.02	**0.14**	0.02	**0.12**	**0.14**	**0.11**	**0.18**	**0.08**	1				
Lab-TAB fear	**0.18**	−0.03	0.01	0.03	0.02	0.02	–	–	−**0.10**	0.07	**0.16**	0.05	−0.05	−0.07	−0.03	−0.01	1			
Lab-TAB anger	**0.17**	**0.22**	**0.19**	−0.03	−0.06	−0.04	0.05	−0.06	–	–	0.39	0.32	**0.08**	0.04	−**0.07**	**0.01**	−0.01	1		
Lab-TAB sadness	−0.12	−0.06	−0.15	0.10	−0.12	0.06	**0.09**	**0.10**	**0.38**	**0.34**	–	–	−0.06	0.03	−0.02	**0.10**	**0.14**	**0.51**	1	
Lab-TAB PA	−0.09	0.05	0.07	0.04	0.02	0.07	−**0.09**	−0.04	**0.18**	−0.06	−**0.09**	0.04	–	–	**0.09**	**0.15**	−**0.11**	**0.07**	−0.03	1

*Bold correlations are significant at the 0.05 level. Ns range from 527 to 878. Due to varying sample sizes, the size of the correlation required to reach significance varies for each scale or episode. IBQ, Infant Behavior Questionnaire; AR, Arm Restraint; PA, Positive Affect; AL, Activity Level; FP, Free Play; Lab-TAB, Laboratory Temperament Assessment Battery. Correlations account for non-independence of twins within family*.

**Table 5:** **Means, standard deviations, and gender comparisons for infant temperament measures**.

	**6-Month**				**12-Month**			
**Temperament measure**	**Total *N***	**Girls mean**	**Boys mean**	**Gender diff. *t*-value**	**Total *N***	**Girls mean**	**Boys mean**	**Gender diff. *t*-value**
IBQ fear	500	2.60	2.48	2.36^*^	709	3.20	3.10	2.02^*^
IBQ distress/anger	494	3.31	3.41	−1.89	705	3.76	3.79	−0.66
IBQ smiling and laughter	500	4.78	4.90	−1.95	713	5.08	5.12	−0.85
IBQ soothability	499	4.88	4.86	0.42	703	4.92	4.91	0.24
IBQ orienting	500	3.66	3.68	−0.36	703	3.66	3.59	0.95
IBQ activity level	500	4.13	4.13	0.08	703	4.34	4.47	−0.02
Fear—stranger	515	0.04	−0.04	1.23	802	0.023	−0.026	1.04
Fear—masks	557	0.05	−0.06	1.91	878	0.043	−0.047	1.87
Anger—arm restraint	537	0.01	−0.002	0.11	826	−0.02	0.02	−0.63
Anger—car seat	499	0.01	−0.001	0.28	775	−0.04	0.04	−1.35
Sadness—arm restraint	536	−0.01	0.01	−0.19	825	−0.03	0.03	−1.08
Sadness—car seat	499	−0.10	0.11	−2.85^**^	775	−0.05	0.04	−1.57
Positive affect—puppets	551	−0.001	0.01	−0.16	867	0.009	−0.008	0.34
Positive affect—peekaboo	534	−0.07	0.08	−2.76^**^	833	−0.045	0.050	−2.11^*^
Slides/interest	536	−0.08	0.06	−2.61^**^	877	−0.048	0.035	−2.11^*^
Activity level (BT and FP)	369	−0.14	0.16	−3.26^***^	837	−0.06	0.06	−2.74^**^
Lab-TAB fear	575	0.06	−0.04	1.99^*^	899	0.033	−0.029	1.67
Lab-TAB anger	564	0.05	−0.01	0.94	878	−0.02	0.04	−1.25
Lab-TAB sadness	563	−0.02	0.04	−1.29	878	−0.03	0.04	−1.57
Lab-TAB positive affect	565	−0.05	0.040	−1.87	894	−0.021	−0.010	−0.84

* p < 0. 05;

** p < 0. 01;

*** p < 0.001;

*IBQ, Infant Behavior Questionnaire; Lab-TAB, Laboratory Temperament Assessment Battery; BT, Basket of Toys; FP, Free Play. IBQ measures are raw scores. Lab-TAB measures are z-scores*.

In the positive affect dimension, no differences in Puppets or overall positive affect at either age occurred, but a significant gender difference did occur for the Peekaboo episode. Boys were rated higher in positive affect coded from the Peekaboo episode of the Lab-TAB at both 6 and 12 months. Boys were scored higher in interest/persistence as measured by the Slides episode and in activity level across time (see Table 5).

Because we standardize the Lab-TAB raw data prior to creating episode scores, and are unable to examine mean level differences in observed temperament. However, we can look at rank order stability across time. Bivariate correlations indicated longitudinal (rank-order) stability in both the parent reported and observed measures of temperament; see correlations across time in Table 6 (left side of the table). The stabilities of IBQ scales (mother + father averages) ranged from 0.41 to 0.59. Although the stabilities of observed behavior reflected in the Lab-TAB composites was substantially lower than the questionnaire scales, only one Lab-TAB episode, Car Seat (Anger), did not show significant longitudinal stability between 6 and 12 months of age. As expected, the cross-episode dimension composites (last 4 rows of Table 6) were more stable than the single episode measures.

**Table 6:** **Cross-time (rank order) stability for infant temperament measures**.

		**Cross-time (6→12 month) rank order stability**			
**Temperament measure**	***N***	**Overall *r***	**Girls *r***	**Boys *r***	**Gender comparison *r-* to *-z***
IBQ fear	384	0.41^***^	0.33^***^	0.45^***^	−1.38
IBQ Distress/anger	376	0.59^***^	0.56^***^	0.54^***^	0.28
IBQ smiling & laughter	388	0.58^***^	0.56^***^	0.54^***^	0.28
IBQ soothability	388	0.44^***^	0.42^***^	0.41^***^	−0.12
IBQ orienting	388	0.58^***^	0.58^***^	0.56^***^	0.29
IBQ activity level	388	0.52^***^	0.46^***^	0.51^***^	−0.64
Fear—stranger	390	0.26^***^	0.31^***^	0.18^*^	1.42
Fear—masks	476	0.15^**^	0.18^**^	0.08	1.04
Anger—arm restraint	438	0.11^*^	0.18^**^	0.02	1.66
Anger—car seat	370	0.05	0.06	0.05	0.10
Sadness—arm restraint	438	0.21^**^	0.18^**^	0.25^***^	−0.75
Sadness—car seat	370	0.31^***^	0.13	0.42^***^	−3.24^**^
Positive affect—puppets	455	0.17^***^	0.24^***^	0.09	2.13^*^
Positive affect—peekaboo	424	0.10^*^	0.14^*^	0.02	1.24
Slides/interest	452	0.13^**^	0.13	0.10	0.31
Activity level (BT and FP)	301	0.38^***^	0.28^***^	0.48^***^	−2.40^**^
Lab-TAB fear	497	0.20^***^	0.22^**^	0.14^*^	0.85
Lab-TAB anger	479	0.14^**^	0.14^*^	0.16^*^	−0.21
Lab-TAB sadness	479	0.32^***^	0.20^**^	0.44^***^	−2.75^**^
Lab-TAB positive affect	481	0.19^***^	0.22^***^	0.14^*^	0.85

* p < 0. 05;

** p < 0. 01;

*** p < 0.001;

*IBQ, Infant Behavior Questionnaire; Lab-TAB, Laboratory Temperament Assessment Battery; BT, Basket of Toys; FP, Free Play. Correlations account for non-independence of twins within family*.

Regarding the stability of temperament across gender, correlations between 6 and 12 months for are presented on the right side of Table 6. We first correlated infant temperament measures across time for girls and boys, and then transformed the Pearson *r* values to *z*-scores to compare the degree to which stability existed across gender. For parent reported temperament, significant stability was observed for each scale, and this stability did not differ by infant gender. For Lab-TAB, boys exhibited significantly greater stability than girls in sadness and activity level, indicated by significant *z*-scores for Car Seat sadness, activity level, and the overall sadness dimension.

## Discussion

The Lab-TAB is a useful tool to measure an infant's tendency to respond to situations with emotional incentives. By exposing infants to the same stimuli in a structured laboratory setting, researchers gain control that questionnaires cannot afford. Here, we describe the theoretical background as well as psychometric properties of the Lab-TAB using an existing sample of more than 1,000 infants. It is not our aim to prescribe the Lab-TAB as a fixed entity, nor to provide rigid rules for tis use. Instead, we illustrate the Lab-TAB's utility across multiple systems. We support its development by other researchers who have used Lab-TAB episodes with different coding and data reduction approaches (e.g., Dyson et al., 2012, 2015). We leave it to each researcher to incorporate those episodes that best fit the researcher's hypothesis but we encourage researchers to consider assessing multiple temperament dimensions using multiple episodes.

### Dimensional vs. episodic LAB-TAB usage

Although fine grained measurement of temperament is informative, researchers often examine infant behaviors in terms of much broader factors such as positivity or surgency, negative affect, and regulation (Putnam et al., 2001). In our study, within measurement method, temperament scales indicative of broader positivity, negativity, and regulatory dimensions were related. That is, infant smiling and laughter, or positive affect, was inversely related to negative dimensions such as fear; fear, and anger were positively related at a given age; and soothability and orienting were positively related. In addition, similar to Bridges et al. (1993), anger and orienting were inversely related.

When incorporating Lab-TAB assessment of infant behaviors, some research questions may pertain to an infant's discrete response to a specific situation, for example, a tendency to respond by struggling to distressing goal blockage such as in the Arm Restraint episode. The use of a single episode from the Lab-TAB may be sufficient to incorporate into such studies. However, to examine inter-relationships among temperament constructs, multiple episodes are obviously needed, and multiple episodes within a domain, such as anger, are needed to claim cross-situational generality. In our study, we first determined scores for individual laboratory episodes by calculating the mean and peak of each infant behavior. In this way, we consider an infant's characteristic reaction throughout the relevant stimulus exposure (mean of response) as well as the most extreme behavior exhibited (peak).

Additionally, temperament theory intimates that temperament reflects an individual's *propensity* toward emotional reactions; that is, individuals' tendency to respond similarly across multiple situations that share affective incentive characteristics. Accordingly, we also created composite scores to reflect broad (cross-response modality, cross-episode) temperament propensities. Scores from each of the two episodes chosen within each temperament dimension (i.e., fear, anger) were correlated at each age, indicating that the episodes designed to represent a temperament composite elicited similar behaviors from infants. These correlations, however, were only modest, indicating potent situational effects.

In behavioral research, numerous factors typically affect observed behaviors. The adult personality literature offers support for moderate cross-situational consistency of behavior, with many caveats and qualifications (Kenrick and Funder, 1988). Modest cross-episode convergence in the infant version of Lab-TAB may be due to multiple sources. First, infant temperament traits may be a weak organizer of behavior, relative to the situational differences between episodes with apparently similar affective incentives. Second, relative to adults, infants possess a less differentiated sense of self, less developed patterns of affective and self-regulation, and no developed social attitudes. Each of these features of personality may support temperament differences. Third, contextual or incentive differences between episodes may alter the patterning of individual differences in temperament. Or, stated another way, temperament traits may be narrower than researchers tend to believe; for example, “anger proneness” might be too broadly conceptualized. Fourth, state variables in infancy, such as hunger or tiredness, may be powerful enough to obscure temperamental differences. Fifth, the novelty of laboratory context is constant, in a sense, across all Lab-TAB episodes, and this novelty may also obscure temperamental differences that would be apparent in more familiar contexts. Thus, the finding that behaviors across episodes are only modestly correlated should not be surprising. Despite these competing influences on behavior, some evidence of cross-situational consistency in Lab-TAB responses is apparent.

### Convergence between laboratory and parent report of infant temperament

Although most of what we know about infant temperament comes from research using parent reports, laboratory based assessment can elucidate patterns of emotional reactivity during specific situations. Parent reports and observed behavior are inconsistently correlated, and several investigators have elaborated potential reasons for this inconsistency (Rothbart and Goldsmith, 1985; Goldsmith and Hewitt, 2003; Saudino, 2003; Stifter et al., 2008). In a previous report on scale development and validation of the Preschool Lab-TAB (Gagne et al., 2011), laboratory-based assessments of fear, shyness, activity level and inhibitory control showed low to moderate correlations with corresponding mother- reported temperament dimensions. We and others (e.g., Gartstein and Marmion, 2008; Braungart-Rieker et al., 2010) found moderate convergence between parent reported and laboratory observed infant fear and anger, but not positive affect (Gartstein and Marmion, 2008) or interest/persistence (Matheny and Wilson, 1981; Carranza Carnicero et al., 2000).

The reason for discrepant findings for different aspects of temperament might be that infants and young children actually do exhibit distress across the home and laboratory environments more consistently than they express positive affect and interest/persistence in the two types of environment. Alternatively, parents may be more attuned to interpersonally disruptive infant distress or anger (Huebner and Izard, 1988) than to other less obtrusive temperamental traits. Thus, plausible reasons exist for lack of convergence between seemingly corresponding IBQ and Lab-TAB assessments in some cases. Nonetheless, by considering both observed and parent measures of temperament, we gain a clearer picture of the development of multiple temperament dimensions across infancy. Moreover, the pattern of significant correlations of IBQ by Lab-TAB findings in Table 4 is fairly systematic and sensible. For instance, fear is perhaps the most salient aspect of temperament to parents. Parents' IBQ Fear reports are related to a variety of the more objective Lab-TAB measures, a finding that suggests parents may over-assimilate infant behaviors to their own version of a fear/inhibition/shyness concept, which is broader than our concept of fear. An alternate interpretation of this finding is that the novelty of the laboratory context affects the assessment of multiple dimensions of temperament. Also, we observe cross-method correlations between activity level and positive affect, suggesting a role for approach motivation in structuring these correlations.

### Stability and gender differences within temperament dimensions

Consistent with previous literature (Else-Quest et al., 2006) we found gender differences in parent reported and observed temperament, such that girls were higher in the fear dimensions of temperament, and boys were higher in approach related behaviors such as positive affect and activity level.

Further, few studies use laboratory measures of infant temperament when assessing continuity. In ours and others' research, infants' expressions of fear, anger, and positive affect as well as activity level increase from 6 to 12 months (Carranza Carnicero et al., 2000; Rothbart and Bates, 2006; Planalp et al., 2016); however, less is known about infant sadness and interest/persistence. We found rank order stability of temperament across time, reflected by longitudinal correlations within scale, episode, or dimension.

Although our results indicate significant stability within temperament dimension, temperament can be less stable across developmental stages, such as the widely implicative transition at about age 8–9 months (Emde et al., 1975). For example, infants who express more distress at 6 months are not necessarily higher in anger or fear by school age (Lemerise and Dodge, 2008). Cognitive and motor development may impact the ways in which individuals can express emotion. Thus, although temperament is relatively stable within developmental periods such as mid-infancy we are not able to predict stability across longer periods of time. We discuss findings within each temperament dimension below.

#### Fear

As infants become more aware of their surroundings and develop expectations for their safety and security, expressions of fear become progressively more apparent between 6 and 12 months of age. Fear in the presence of a stranger emerges, on average, around 8 months of age and increases through the first year, although substantial individual differences exist in developmental trajectories of stranger fear. Parents rated girls higher than boys on fear, and girls exhibited more overall fear than boys at 6 months, consistent with previous research indicating gender differences in parent-reported fear (Else-Quest et al., 2006; Gartstein et al., 2010). In addition, although girls' observed fear was fairly stable from 6 to 12 months and boys' fear was less so, longitudinal correlations did not significantly differ by gender. According to social learning theory, girls learn to be more wary of environmental novelty and discomfort than boys (Kochanska, 1993; Chaplin and Aldao, 2013); thus, initial socialization processes for both parents and infants might account for our findings for gender differences in the development of fear (Malatesta and Haviland, 1982; Kochanska, 1993; Chaplin et al., 2005; Chaplin and Aldao, 2013).

#### Anger

Similar to previous research (Else-Quest et al., 2006), we did not observe differences between boys' and girls' anger, regardless of measurement method. However, rank order stability was apparent in how infants expressed anger in early infancy, with stronger stability within episode for girls than boys (though this was not significantly different) during the Arm Restraint episode. Comparable to fear responses, anger seems to peak and then dissipate as infants age; by age 3 years, most children do not become overtly angry in the lab-based anger assessments (Gagne and Goldsmith, 2011). The emergence of self-regulation may allow children to better regulate their negative affect; they begin to show more individual differences in expressions of distress which decrease as regulation increases (Gagne and Goldsmith, 2011).

#### Sadness

Boys were higher in Car Seat Sadness at six, but not 12, months. In addition, although all infants' expressions of sadness during Arm Restraint were stable across time, boys exhibited stability in Car Seat sadness whereas girls did not. At 6 months of age, girls might already be beginning to regulate their expressions of distress to limitations better than boys, or alternatively, girls might be more variable in how they express distress to limitations—sometimes withdrawing and other times approaching—resulting in lower stability in sadness. Other than Car Seat, individual differences in observed infant sadness were relatively stable across time and, similar to previous studies concerning fear (Goldsmith and Lemery, 2000). Perhaps their common association with motivation to withdraw accounts for the links between sadness and fear (Buss and Goldsmith, 1998).

We also found that anger and sadness measures from the same episodes were substantially correlated. Anger and sadness were the only affects that were not measured independently, because we were unable to devise ethical (non-harsh) episodes involving loss or goal blockage that reliably elicited sadness but no anger, or vice-versa. The result of this inability is that our estimates of anger by sadness inter-correlations are confounded by their common context of assessment. Perhaps whether infants react to loss or goal blockage with a preponderance of anger vs. sadness is an interesting individual difference in and of itself.

#### Positive affect

Individual differences in infant positivity are relatively stable during infancy and early childhood (Lemery et al., 1999; Bridgett et al., 2013; Planalp and Braungart-Rieker, 2015). In general, girls exhibit more positive affect than boys (Else-Quest et al., 2006). Early in life, however, boys and girls do not show significant differences in positive affect, although boys tend to show more behaviors related to exuberance and activity level by 5 years of age (Degnan et al., 2011). In our study, boys exhibited more positivity during the Peekaboo episode, which involves anticipation and excitement (possibly exuberance) upon seeing the mothers face. Girls, however, showed stronger stability across time in the Peekaboo episode, indicating that perhaps girls are less variable in how they respond to positively valenced stimuli. Nonetheless, our measurement of positive affect reflects the underlying stability evident in many temperamental dimensions.

#### Interest/persistence

Infant interest/persistence is stable starting in the second year of life (Rothbart et al., 2000; Auerbach et al., 2004), but results regarding stability from the first year of life are mixed. Some studies find moderate relations in parent ratings of interest or orienting across ages 3 to 12 months (Matheny and Wilson, 1981) whereas others find no relation when assessment intervals are 6 months or greater (Carranza Carnicero et al., 2000). In addition, observed measures of interest tend to be uncorrelated until later in infancy (Carranza Carnicero et al., 2000). These findings are likely a consequence of the differential development of two attentional systems, an orienting system and a more voluntary system subsuming inhibition and planning (Ruff and Rothbart, 2001). Why boys show more interest/persistence during the Slides episode than girls is unclear. Attentional differences are not typically reported until later in development (Putnam et al., 2001), and parents did not report differences in duration of orienting on the IBQ.

#### Activity level

Similar to previous research, boys were higher in activity level during tasks designed to elicit active responses than girls (Chaplin and Aldao, 2013). Boys may have higher reactive tendencies, evidenced by higher scores on approach or externalizing domains (exuberance, anger) as well as lower regulation in childhood (Weinberg et al., 1999; Degnan et al., 2011; Gagne and Goldsmith, 2011). In addition, we confirmed prior findings that boys were more active than girls in the laboratory (Campbell and Eaton, 1999; Else-Quest et al., 2006). Boys tend to be higher on approach based behaviors such as exuberance and activity level (Weinberg et al., 1999).

Additionally, activity level tends to be stable, such that more active infants are also more active as older children. A meta-analysis of longitudinal studies of temperament showed stability for individual differences in activity level from infancy to childhood (*r* = 0.28, *p* < 0.05; Roberts and DelVecchio, 2000), and in a study of children ages 2 to 3, parent-report and observer ratings evidenced both continuity and change across age (Saudino, 2012). We found similar stability in activity level across infancy in both IBQ and Lab-TAB assessment.

### Limitations and conclusions

Although we conclude that the Lab-TAB offers numerous advantages over subjective parental report, several limitations are also noteworthy. First, administering the full Lab-TAB battery, which we did not do in this study, is time-consuming and expensive, especially when the episodes are repeated to assess stability. Even with the 20 episodes described in the locomotor version of the Lab-TAB manual, we still only sample limited components of the infant's behavioral repertoire and thus only a limited range of affective individual differences. For instance, we do not tap the affective dimension of disgust. Also, for ethical reasons, we do not use more intense elicitors of negative affect that occasionally occur in daily life.

Another set of limitations is characteristic of most laboratory assessments in the social, affective, and even cognitive domains. Examiner differences, scorer sensitivity effects, infant state effects (hunger, tiredness), effects of the novel laboratory environment, and other subtle effects may impact results. We also examined convergent validity of the Lab-TAB episodes and dimensions with parent reported temperament, but did not address discriminant validity between temperament dimensions. Although we considered discriminant validity in the construction of Lab-TAB, we did not consider it in this paper. The issue of discriminant validity, particularly as it applies to Lab-TAB measurement of anger and sadness issue is complex. As explained above, we score anger and sadness from the same episodes, and anger and sadness are correlated due to the common element of measurement, much as questionnaire items on the IBQ that follow from the same stem are correlated.

More generally, formulations about discriminant validity often occur in the absence of theory that specifies how latent constructs should be related. We posit that temperament is affective individuality, so we must use affect theories to offer expectations about discriminant validity. For instance, popular circumplex theories of affect (e.g., Plutchik, 1980) specify that affects at opposite poles of the circumplex would be uncorrelated, but those nearby on the circumplex would be highly correlated. Other theories of affect generate different expectations for the association of components of affect and thus for expectation of what would constitute evidence of discriminant validity. Given these complications, we did not address discriminant validity comprehensively in this manuscript although we do so in more limited contexts in other papers (Goldsmith and Gagne, 2012; Clifford et al., 2015).

Despite these limitations, we provide more support and validation for the use of an infant laboratory based temperament assessment. The Lab-TAB indicated moderate relations with parent reports, consistency across time, and replicated gender differences from prior research. In addition, we provide information on the use of both episode-level vs. dimensional scoring, which allows researchers flexibility depending on their question of interest.

We conclude that employing the pre-locomotor and locomotor versions of the Lab-TAB as a behavioral assessment of temperament in infancy is feasible and fruitful for examining temperament using an individual differences approach. Researchers will vary on administration (single episodes for each temperament dimension vs. multiple episodes), coding and scoring (e.g., some will prefer to use more global scoring than we did, cf. Dyson et al., 2012). Lab-TAB can be used to examine temperament within age, longitudinally across infancy (as we did) and into childhood, and within and across temperament dimensions. In sum, the Lab-TAB is a useful tool for examining fine grained individual differences in early emerging infant temperament.

## Author contributions

EP conceived the paper, ran statistical analyses and contributed to the manuscript. CV did statistical analyses and contributed to the manuscript. JG did statistical analyses and contributed to the manuscript. HG conceived the paper and contributed to the manuscript.

### Conflict of interest statement

The authors declare that the research was conducted in the absence of any commercial or financial relationships that could be construed as a potential conflict of interest.
